# Plasma Modification Effects of Thermoplastic Starch (TPS) Surface Layer: Film Wettability and Sterilization

**DOI:** 10.3390/ma18092156

**Published:** 2025-05-07

**Authors:** Magdalena Stepczyńska, Aleksandra Śpionek

**Affiliations:** Department of Materials Engineering, Kazimierz Wielki University, Chodkiewicza 30, 85-064 Bydgoszcz, Poland

**Keywords:** thermoplastic starch film, low-temperature plasma, corona discharge, wettability, surface layer, sterilization, plasma surface modification, surface treatment

## Abstract

The effect of low-temperature plasma treatment on the surface properties of thermoplastic starch film (TPS) was investigated. The surface layer (SL) modification of polymeric materials is mainly carried out to improve wettability and adhesive properties and to increase surface cleanliness. TPS was modified in an air atmosphere under either atmospheric or reduced pressure. The process parameters for modifying the SL of TPS were determined based on wettability assessment using a goniometer, geometric structure using scanning electron microscopy (SEM), and the degree of oxidation (O/C ratio) using X-ray photoelectron spectroscopy (XPS). Additionally, the effect of plasma treatment on TPS film sterilization was investigated.

## 1. Introduction

Economic growth and continuous technological advancements have led to an increasing demand for polymer-based products with novel properties. Consequently, the volume of polymer waste is steadily rising, posing significant environmental burdens and contributing to widespread pollution. An awareness of the environmental risks associated with plastic waste, along with EU directives and the depletion of petroleum resources, has driven increased interest in biodegradable materials.

Due to their biocompatibility and biodegradability, biodegradable polymers have been utilized in medicine since the 1960s. Over the past decade, their applications have expanded into the packaging, pharmaceutical, biotechnology, and horticulture industries, primarily for the production of food and drug packaging, as well as medical instruments.

Thermoplastic starch (TPS) is a biodegradable material derived from natural starch sources such as corn, potato, or cassava [1,2]. Through plasticization—typically involving glycerin, glycerol, and other plasticizers—starch granules are disrupted, resulting in a thermoplastic material that can be molded and processed similarly to conventional polymeric materials [3,4,5,6,7]. Due to its biodegradability, renewability, and cost-effectiveness, TPS has garnered significant attention as an alternative to petroleum-based plastics, particularly in packaging applications [1,2,8,9,10,11,12]. A crucial prerequisite for the use of TPS and other biodegradable materials in such applications is the proper preparation of their surface layer (SL) for printing, bonding, or decorating, as well as ensuring sterilization, given that packaging must be free from microorganisms [13,14].

In recent years, there has been a significant surge in interest in the surface engineering of biodegradable materials. Wherever these materials come into direct contact with other substances (including other polymers), their surface properties play a crucial role. By employing appropriate surface modification techniques, biodegradable materials can be endowed with entirely new properties or improved surface characteristics. Surface modification is primarily conducted to enhance adhesion, increase cleanliness and surface roughness, and improve wettability [15,16,17,18,19,20,21].

Several techniques for modifying the surface layer of polymeric materials are known, including mechanical, chemical (both dry and wet), flame, and plasma methods. However, not all of these are suitable for modifying biodegradable polymers, such as thermoplastic starch (TPS), due to their inherent properties. The choice of modification techniques must also consider the sustainability aspect of biodegradable polymers. Given the need to minimize environmental impact, the use of chemical modification methods or strongly oxidizing liquids is not desirable.

The surface modification of TPS using corona discharge (CD) or low-temperature plasma under reduced pressure represents an economical and effective approach to altering its surface properties. These techniques allow for the retention of key biomaterial characteristics, such as strength and biocompatibility, while enhancing biofunctionality. Furthermore, they are environmentally safe and do not pose a threat to the modified biodegradable material itself. These methods enable precise alterations to the surface layer of TPS films, particularly in terms of wettability, surface free energy (SFE), and adhesion. Such modifications result from the incorporation of polar functional groups, primarily through oxidation processes, increased polymer crosslinking, and enhanced surface roughness [15,18,22,23,24,25].

Corona discharge is the most widely used industrial technique for modifying the surface layer of polymers. It is commonly applied in the plastic industry, including the biodegradable packaging industry, to alter surface properties and facilitate sterilization. Low-temperature plasma modification has been extensively studied since the 1950s and is utilized in various industrial sectors where modifications of the chemical and physical structure of the surface layer are required, as well as for sterilizing food packaging, including biodegradable materials, and medical equipment [26,27,28,29,30,31,32,33]. These methods are highly advantageous as they do not affect the bulk properties of the material beneath the modified layer. Additionally, both processes are straightforward, easily controllable, and environmentally safe [34,35].

However, to successfully transition from laboratory research to industrial-scale application for TPS, it is crucial to develop scalable plasma reactor systems. Future efforts should focus on optimizing continuous or roll-to-roll plasma systems that provide uniform surface treatment for TPS, enhance energy efficiency, and integrate smoothly into existing production lines. Additionally, attention should be given to reactor geometry, electrode configuration, and process control systems to ensure consistent treatment reproducibility at higher throughput levels [36,37,38].

Currently, gamma radiation and chemical disinfection methods are commonly used for the sterilization of medical equipment and food packaging. Unfortunately, biodegradable materials, including TPS films, are not resistant to these factors and, in addition, the chemicals used may alter their properties and durability. Moreover, residues of disinfectants present on the surface of biodegradable packaging may have a significant impact on human health. Therefore, the use of plasma modification, which exhibits biocidal activity and, at the same time, does not adversely affect the physical and chemical properties of the TPS film, is justified. In contrast, plasma treatment improves the surface properties of the TPS film and positively affects wettability, surface free energy (SFE), and adhesion. Corona discharge and low-temperature plasma under reduced pressure, used to improve the surface properties and sterilize TPS films, will not be harmful to consumer health and are environmentally safe [39,40,41,42,43].

The exploration of new possibilities for modifying the surface layer of TPS and the need for its sterilization were the primary motivations behind this research in the field of materials engineering. Therefore, the present study focuses on selected aspects related to enhancing the surface properties of TPS films, particularly their potential for plasma-based sterilization.

## 2. Materials and Methods

The composites were primarily composed of potato starch from the Potato Industry Company (Trzemeszno, Poland). Glycerine (PolAura, Zawroty, Poland), with a molecular weight of 92.1 g/mol, functioned as the plasticizer.

The mass ratio of starch to glycerine was set at 75/25. The production process began with drying the starch in a laboratory dryer (SUP-100 G, Wamed, Poznań, Poland) at 60 °C for 24 h to remove moisture, a necessary step due to the starch’s hygroscopic nature. Subsequently, the precisely measured components were thoroughly mixed until a homogeneous mass was achieved. The resulting material was processed into TPS film using a twin-screw extruder (BTSK 20/40D, Bühler, Braunschweig, Germany). The temperatures of the individual heating zones of the extruder were 95 °C (I), 100 °C (II), 100 °C (III), and 100 °C (IV). The temperature of the extruder head was 100 °C. The rotational speed of the extruder screw was 35 min^−1^. TPS film was about 120 μm thick.

The TPS film was modified using the AF2 film activator (IPTS Metalchem, Toruń, Poland) and a Femto plasma generator (Diener Electronic GmbH, Ebhausen, Germany) with a nominal power of 100 W.

The main characteristics of the corona activator include a generator with a nominal power of 2 kW (Energoelektronika, Bydgoszcz, Poland), a discharge frequency of 50 kHz, an inter-electrode voltage of 14 kV, a single-tip high-voltage electrode (HVE) for discharges in air (0.25 m long, made of aluminium due to its resistance to the oxidizing effect of ozone), an earthed electrode (EE), an inter-electrode gap adjustment accuracy of 0.1 mm, and a film feed velocity of 0–100 m/min. Due to the TPS film thickness of 0.15 mm, the distance between the HVE and EE was 1.9 mm. The width of the inter-electrode gap should be as small as possible to ensure uniform corona modification.

Corona discharges occur due to a potential difference established between two electrodes of the activator: the high-voltage discharge electrode and the grounded electrode, within a gas-filled space, typically atmospheric air at ambient pressure. Plasma formation is initiated by free electrons in the interelectrode region, which, under the influence of an electromagnetic field, undergo significant acceleration. As these electrons move through the interelectrode space, they collide with air molecules, inducing ionization and increasing the population of electrons and ions. As a result, an electric current begins to flow within this region. The plasma stream directed onto the material’s surface layer effectively removes contaminants while simultaneously modifying its geometric structure [30,44,45,46,47,48].

These high-energy electrons, upon impact with the material, break certain chemical bonds, leading to the formation of reactive radicals. These radicals trigger chemical reactions, primarily oxidation processes, by interacting with oxygen, ozone, hydroxyl groups, and water molecules, leading to the formation of polar compounds [49].

Through oxidative decomposition reactions, macromolecules containing polar functional groups are generated within the surface layer, thereby altering the material’s surface properties—most notably, increasing its surface free energy (SFE) [17].

The extent of the modification of the TPS film surface layer was determined by the unit energy (E_j_) of modification. The E_j_ value is a basic factor that affects changes in the surface layer of the modified TPS.

TPS film modified by the corona treatment in the air over the E_j_ range of 1–15 kJ/m^2^ (Table 1). The process was performed at ambient temperature (about 23 °C) and under atmospheric pressure, using the E_j_ values determined from the following formula:

(1) $$E_{j}=\frac{P}{L\cdot v}$$

where P—the power of the corona discharges occurring in the inter-electrode gap of the activator (P = 0.4 kW); L—high-voltage electrode length (L = 0.25 m); v—transfer velocity of the modified specimens.

Samples of TPS modified by low-temperature plasma were successively placed in the generator vacuum chamber on a metal slab and exposed to the effect of a plasma discharge generated in air under lowered pressure (20 Pa). The samples were modified with a plasma power of 20, 35, and 40 W for 3, 10, and 15 min. Sample symbols and modification conditions are given in Table 2.

Low-temperature plasma used to modify WW materials is generated under the influence of a rapidly changing electromagnetic field in a vacuum chamber, called a discharge chamber, where the gas pressure is from 0.05 to 5 hPa. The average temperature in the chamber is close to the ambient temperature, while the electron concentration in the plasma is in the range of 10^16^ to 10^18^ ē/cm^3^. The electromagnetic field is generated between two electrodes located in the discharge chamber. A high-frequency electrical voltage is supplied to the electrodes [30,32].

The plasma formation process is also accompanied by electromagnetic radiation, including UV. The source of this radiation is excited atoms or gas molecules. Its intensity depends on the type of gas in which the discharges occur, the pressure, and the power of the discharge device.

A goniometer (DSA 100, Krüss GmbH, Germany) equipped with an automated droplet dosing system was used to measure the contact angle. The examination has been conducted according to the ISO 8296 standard [50]. Measurements were performed using water and diiodomethane as test liquids. Droplets were placed at the center of the tested TPS film surface, with the droplet volume continuously increased while simultaneously recording the dynamic contact angle. To minimize the effect of gravity, which deforms the spherical shape of diiodomethane droplets—whose specific gravity is approximately three times that of water—smaller diiodomethane droplets, with a volume nearly three times lower than those of water, were used in the experiments. Table 3 presents the range of droplet volume (V) variations, the flow rate (∆V), and the time interval (τ) between contact angle measurements for each test liquid.

For each TPS film, 12 contact angle measurements were conducted using water and diiodomethane, with the two extreme values (the highest and lowest) discarded. To determine the advancing contact angle, 96 measurements were taken for each water droplet and 45 for each diiodomethane droplet. The number of measurements was based on the droplet volume (V) range and the selected time interval (∆τ) between measurements. The lower number of measurements for diiodomethane resulted from its smaller range of droplet volume variation. Based on the obtained results for each sample, the mean contact angle was calculated.

Based on the contact angle measurements, the surface free energy (SFE) was calculated using the Owens–Wendt method [51], which distinguishes two components of SFE (γ_S_): polar ($\gamma_{s}^{p}$) and dispersive ($\gamma_{s}^{d}$).

(2) $$\gamma_{S}=\gamma_{s}^{d}+\gamma_{s}^{p}$$

A consequence of this division is the assumption that intermolecular attractions occur exclusively under the influence of forces of the same kind. Using Berthelot’s hypothesis, according to which the interactions between the molecules of two different bodies are equal to the geometric mean of the interactions between the molecules of each of these bodies, Owens and Wendt proposed an equation describing the interfacial SFE in the form

(3) $$\gamma_{SL}=\gamma_{S}+\gamma_{L}-2{\left(\gamma_{S}^{d}+\gamma_{L}^{d}\right)}^{1/2}-2{\left(\gamma_{S}^{p}+\gamma_{L}^{p}\right)}^{1/2}$$

From Equations (2) and (3),

(4) $$\gamma_{L}\frac{1+\cos\theta }{2}={\left(\gamma_{S}^{d}\gamma_{L}^{d}\right)}^{1/2}+{\left(\gamma_{S}^{p}\gamma_{L}^{p}\right)}^{1/2}$$

In Equation (4), two unknown variables, $\gamma_{s}^{d}$ and $\gamma_{s}^{p}$, are present. Therefore, to determine their values, it is necessary to measure the contact angle using two different test liquids. This approach yields a system of two equations with two unknowns. For accurate analysis, the pair of test liquids should be selected such that one has a high dispersive component $\gamma_{s}^{d}$ and a low polar component $\gamma_{s}^{p}$, while the other exhibits the opposite characteristics—a high polar component and a low dispersive component. Based on these criteria, water (polar liquid) and diiodomethane (dipersive liquid) were chosen for the study. The surface free energy parameters for water are $\gamma_{s}^{p}$ = 51 [mJ/m^2^] and $\gamma_{s}^{d}$ = 21.8 [mJ/m^2^], for diiodomethane are $\gamma_{s}^{p}$ = 2.3 [mJ/m^2^] and $\gamma_{s}^{d}$ = 48.5 [mJ/m^2^] [52,53].

Using water and diiodomethane as measuring liquids, the SFE was calculated from the system of equations

(5) $$\left\{\begin{array}{c} {\left(\gamma_{s}^{d}\right)}^{1/2}+1.53{\left(\gamma_{s}^{p}\right)}^{1/2}=7.80(1+\cos\theta_{w})\\ {\left(\gamma_{s}^{d}\right)}^{1/2}+0.22{\left(\gamma_{s}^{p}\right)}^{1/2}=3.65(1+\cos\theta_{d}) \end{array}\right.$$

where θ—contact angles.

The Owens–Wendt method is the most common method used in the calculation of the SFE of solid-state polymeric materials [54,55,56].

The oxidation degree (O/C ratio) of the TPS film surface layer was analyzed using X-ray photoelectron spectroscopy (XPS) with an Escalab 210 electron spectrophotometer (VG Scientific, Birmingham, UK). The base pressure in the spectrometer analysis chamber was 2 × 10^−10^ mbar. During the measurements of the described samples, due to the release of volatile particles from their surface, the pressure was maintained at 1 × 10^−8^ mbar. The excitation beam, emitted by the AlKα source (1486.6 eV), was incident on the sample surface at an angle of 55 degrees. The photoelectron energy was recorded using a VG-Scienta R3000 (Scienta Omicron, Uppsala, Sweden) hemispherical analyzer. The spectra were recorded with an energy step of ΔE = 1 meV. The experimental data were fitted to Gauss–Lorentz curves using the CasaXPS software(Version 2.3.16; Casa Software Ltd., Teignmouth, UK).

Images of the morphology of modified SL TPS were recorded by using the scanning electron microscope (SEM) Phenom XL from ThermoFisher Scientific (Eindhoven, The Netherlands). Before imaging, the surface of the tested samples was covered with a layer of gold. The conductive layer was sputtered in MCM100P (SEC, Gyeonggi, Korea) low-vacuum sputtering machine.

To evaluate the sterilization effect of plasma, three TPS film specimens were prepared for each modification power and time as test samples. Additionally, three film specimens were prepared as check samples to determine the number of bacterial cells recovered from the unmodified TPS. Before depositing the microbial material, the PLA specimens were disinfected with 80% ethanol.

The sterilization effect of plasma was evaluated following the ISO 22196:2011 standard [57], which measures antibacterial activity on plastics and other non-porous surfaces. This study used reference bacterial strains: *Staphylococcus aureus* (ATCC 6538P) and *Escherichia coli* (ATCC 8739).

Each culture of a strain was prepared by a standard method of evaluation of optical density of the prepared cell suspension with the use of a densitometer (Densitometer II, PLIVA-Lachema Diagnostika, Brno, Czech Republic) based on the McFarland’s scale [58]. The suspension was found to be ca. 0.5 of the McFarland unit, which corresponded to 1.5 × 10^8^ cells in 1 mL of the suspension. Next, 20 µL of a prepared bacterial suspension, corresponding to the bacterial cell total number of 3 × 10^6^, was placed on each TPS film. The specimens were successively placed in the generator vacuum chamber and modified under an air atmosphere at a pressure of 20 Pa. The TPS surface with the deposited bacteria was the side that was both modified and examined. The degree of reduction (R) of the number of living and viable cells of tested bacteria was calculated using the following equation:

R = (U_t_ − U_0_) − (W − U_0_) (6)

where

U_0_—the logarithm of the number of viable bacteria obtained from the control samples immediately after inoculation;

U_t_—the logarithm of the number of viable bacteria obtained from the control samples after 24 h, serving as a measure of survival over time;

W—the logarithm of the number of viable bacteria obtained from the tested samples after 24 h.

According to standard (ISO 22196, [57]) for the measurement of antibacterial activity on plastics and other non-porous surfaces, a bactericidal agent may be considered as effective if the reduction in the number of cells capable of growth is by two orders of magnitude or more (R ≥ 2).

Surface images of the modified and unmodified films with deposited bacteria were taken using an epi-fluorescence microscope and the LIVE/DEAD test, which enabled differentiation between living and dead cells. In this test, two stains—propidium iodide and CYTO Green (Invitrogen, Eugene, OR, USA)—were applied to the surface of the TPS films. Next, the excess stain was removed, and the films were dried. As a result of this procedure, living bacterial cells appeared green, while dead ones turned red or orange.

The sample of unmodified TPS is symbolized by N. The films modified by corona discharges or low-temperature plasma are symbolized by C or P, respectively. The first number placed after the C or P symbol indicates the unit energy/power modification (1–15 kJ/m^2^ or 20–40 W, respectively), and the second number indicates the modification time (3, 10, or 15 min).

## 3. Results and Discussion

### 3.1. Wettability and Surface Free Energy

**Figure 1:**
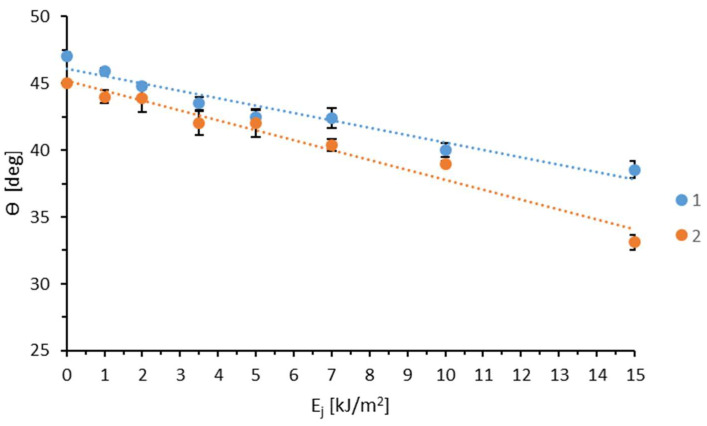
Effect of unit energy (E_j_) on water (1) and diiodomethane (2) contact angle (ϴ).

The results of measurements of the contact angles of water or diiodomethane for TPS film modified by the WK method in an air atmosphere are presented in Figure 1.

The water contact angle decreases as the unit energy of modification increases. The initial value of 47.00° for the unmodified sample declines to 38.55° at the highest energy level (15 J/m^2^). The diiodomethane contact angle also decreases, though in a more irregular pattern. Starting at 45.00° for the unmodified sample, it drops to 33.10° at the highest energy level. The standard deviation ranged from 0.2 to 0.8 for water and 0.2 to 1.1 for diiodomethane.

**Figure 2:**
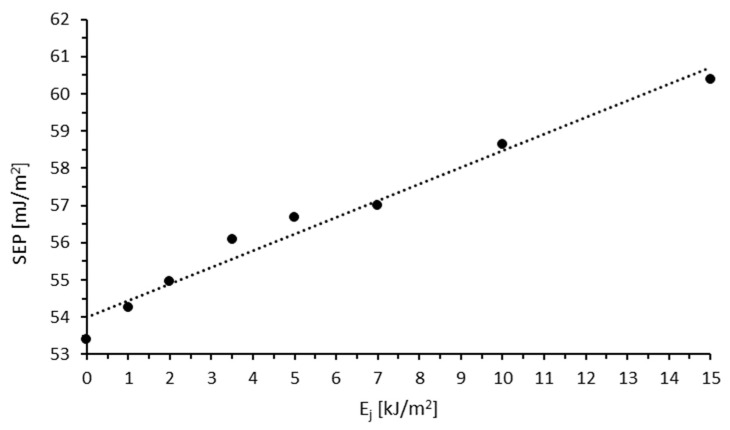
Effect of unit energy (E_j_) on surface free energy (SEP).

This is because polar forces play a more significant role in intermolecular interactions. The considerably greater increase in the interactions of polar liquids, compared to dispersive liquids, with the modified TPS film results from the formation of polar functional groups (such as OH and -COOH) in the TPS surface layer during the modification process. These groups strongly interact with water molecules, whereas dispersion interactions are of lesser importance. Based on the measurements of the contact angles with water or diiodomethane, the SEP was calculated using the Owens–Wendt method (Figure 2).

With the increase in E_j_ value, SEP increases. The changes in the SEP values of TPS film modified in air range from 53.4 to 60.39 mJ/m^2^. The increase in SEP is primarily caused by the rise in the polar component, which results from the formation of polar groups that strongly interact with water molecules and enhance wettability.

**Figure 3:**
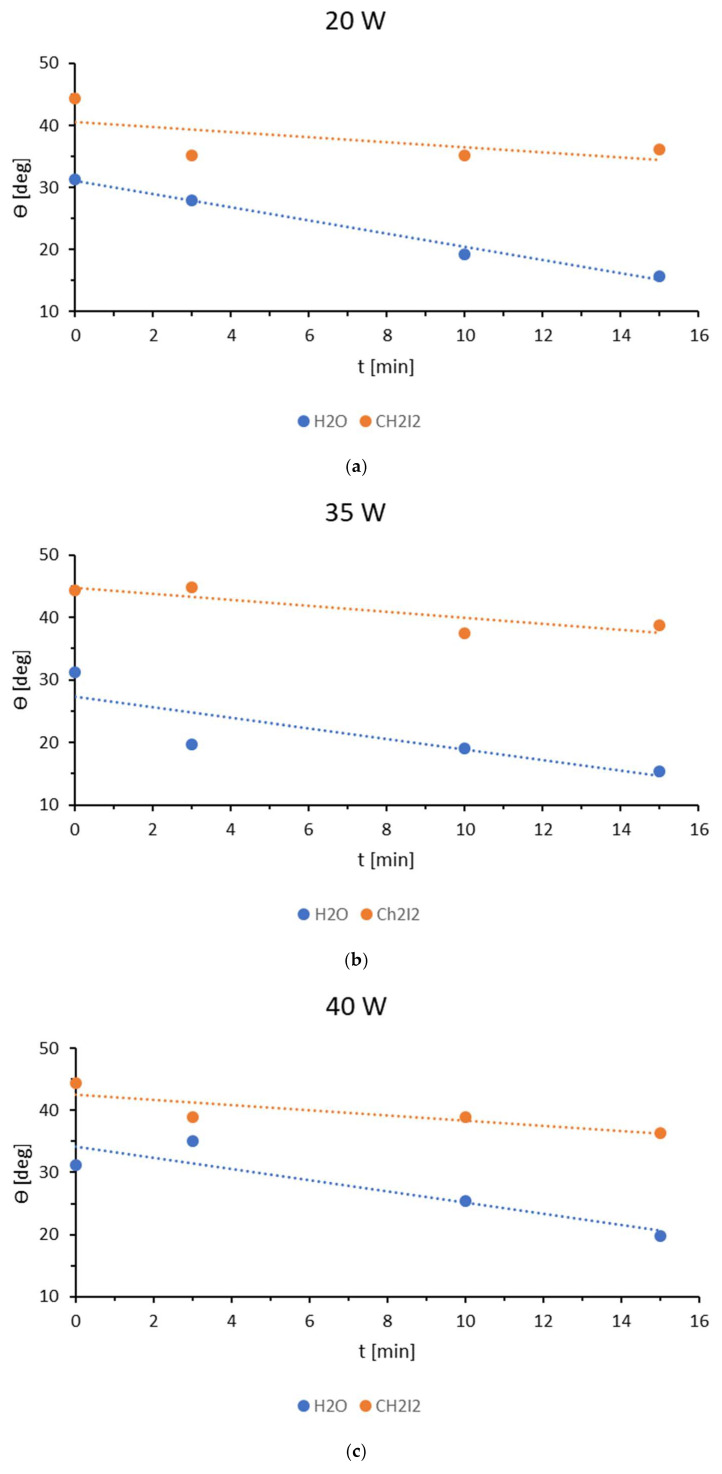
The surface contact angles of the TPS film modified with low-temperature plasma at a power of: (**a**) 20 W, (**b**) 35 W, and (**c**) 40 W.

Analyzing the results of contact angle measurements for TPS film modified with low-temperature plasma at a power of 20 W (Figure 3a), a decrease in their values can be observed for both polar and dispersive liquids. However, the change in the contact angle values for water is much greater than for diiodomethane because polar forces have a greater share in the intermolecular interaction. Modification with low-temperature plasma causes the formation of polar groups in the surface layer that strongly interact with water molecules [studium]. The dependence of water contact angles on modification time is very close to a decreasing linear function. The lowest value of the water contact angle occurs for the longest time used in the study, i.e., 15 min, and is 15.77°. The contact angle for diiodomethane changes differently. The greatest influence on its value has a 3 min modification time. Wettability decreases by 9.1° compared to the unmodified material. The further exposure of the material to low-temperature plasma does not cause significant changes in the measurements of the contact angles of the dispersion liquid: the differences between the results are less than one degree.

The results of wetting angle measurements for water and diiodomethane on TPS foil modified with low-temperature plasma at a generator power of 35 W (Figure 3b) show a downward trend in the water wetting angle as the modification time increases. The greatest decrease in this value is observed during plasma modification up to three minutes, with the water wetting angle decreasing from 31.24° to 19.69°. Increasing the modification time from 3 to 10 min causes only a minimal change in the polar liquid wetting angle, with a decrease of less than 0.6°. However, further extending the modification to 15 min significantly decreases the wetting angle from 19.13° to 15.46°. A different trend is observed for the dispersive liquid. For this liquid, the greatest decrease occurs when the modification time is extended from 3 to 10 min, with a reduction of 7.38°. Further modification results in a slight increase in wettability with diiodomethane, from 37.51° to 38.77°.

Figure 3c shows the results of contact angle measurements for water and diiodomethane on TPS film modified with low-temperature plasma at a generator power of 45 W. An initial increase in the water contact angle to 40.76° is observed after a modification time of three minutes. This increase could be attributed to a measurement error or contamination of the sample’s surface layer. However, as the modification time continues, a gradual decrease in the measured value is observed. The material modified with plasma for 10 min shows a wettability of 25.43°, while, after 15 min of modification, the contact angle drops to values below 20°. The diiodomethane contact angle decreases with increasing modification time, reaching 36.05° after 15 min.

**Figure 4:**
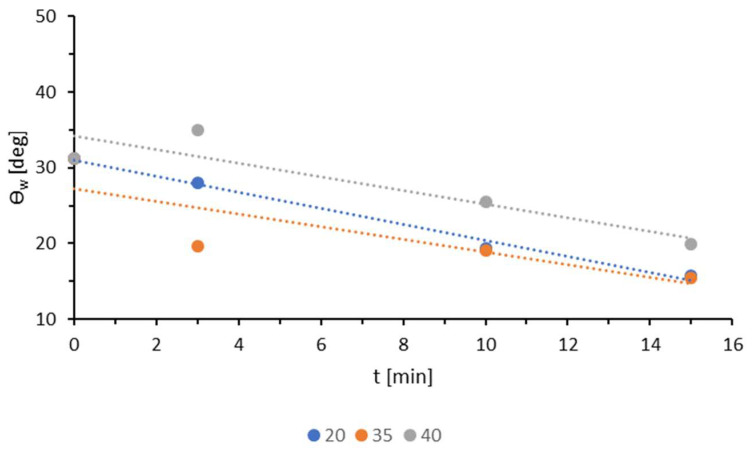
The water contact angles for different generator powers.

Analyzing the results for all applied modification powers, it is evident that low-temperature plasma has a greater influence on the contact angles of water than on those of diiodomethane. For each power used, the change in wettability for the polar liquid was more pronounced. As previously mentioned, during modification with low-temperature plasma, polar groups are formed that strongly interact with water molecules, thereby increasing the wettability of the top layer of the TPS film. To facilitate the comparison of water contact angles, they are presented in a collective graph in Figure 4. The smallest contact angles for the polar liquid at each tested time were observed for the sample modified with 35 W. This power also shows the sharpest decrease in the contact angle during the first three minutes of modification. Similar effects were observed for 20 W and 35 W with modification times of 10 and 15 min. However, for 20 W, the decrease in the contact angle is more linear. In these cases, the measurement results differ by a maximum of 0.4°.

**Figure 5:**
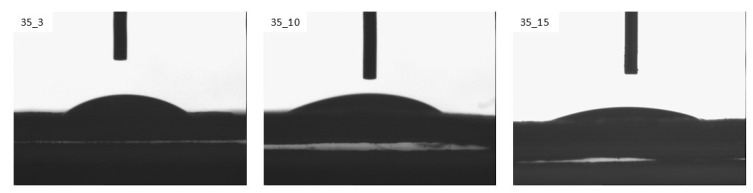
Images of the measuring droplet for the TPS film modified with a 35 W power generator.

The optimal technological parameters for the low-temperature plasma modification of the TPS film are a generator power of 35 W and a modification time of 3 min, as these conditions result in the sharpest increase in wettability. During the measurements, photos of water droplets deposited on the modified samples were taken. Figure 5 shows sample images of the measuring droplet for the TPS film modified with plasma under reduced pressure using a 35 W power generator. As the modification time increases, the shape of the measuring liquid droplet becomes flatter.

**Figure 6:**
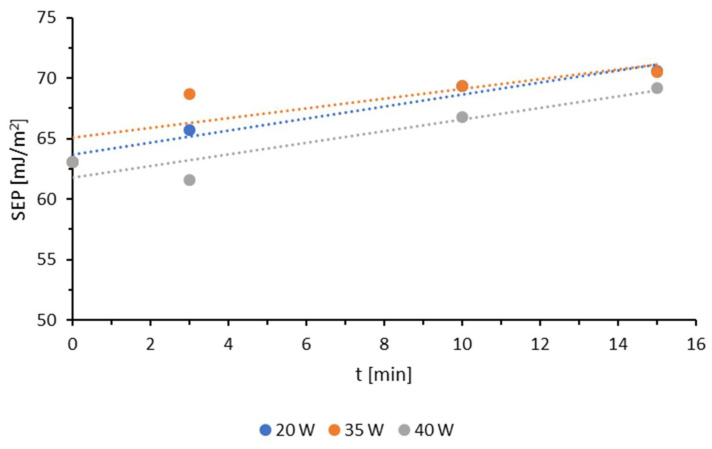
SFE for different generator powers.

Based on the measurements of the contact angles with water or diiodomethane, the SEP was calculated using the Owens–Wendt method (Figure 6).

Plasma modification under reduced pressure resulted in an increase in the surface free energy (SFE) with increasing modification time. An exception was observed for the material modified at a plasma power of 40 W, where a decrease in SFE occurred during the initial 3 min. This phenomenon was likely associated with an increase in the water contact angle under these specific modification parameters, potentially due to sample contamination, probably already at the extrusion stage. Since the contact angle study was conducted each time under the same conditions (temperature, humidity) and methodological safeguards (samples were cut precisely, without touching or rubbing the surface being modified and tested; when sampling, care was taken that the surfaces to be tested did not come into contact with any other material), according to the ISO 8296 standard [50], this is the only reason in this case.

For all applied power levels, the most pronounced changes in SFE were recorded within the first three minutes of treatment. The further extension of the modification time did not lead to a significant increase in SFE, particularly in the 10–15 min range. The TPS film modified at 20 W for 10 min exhibited an SFE of 69.3 mJ/m^2^, whereas prolonging the modification by an additional 5 min resulted in a marginal increase of only 1.24 mJ/m^2^. This effect is likely attributable to the saturation of the surface layer with polar functional groups, which enhance the sample’s wettability. The modification effects at 20 W and 35 W were highly comparable within the 10–15 min treatment period. The highest SFE value recorded in this study was 70.59 mJ/m^2^, observed at a modification time of 15 min and a generator power of 20 W. However, the most rapid increase in SFE was noted within the first 3 min of modification at 35 W.

The increase in SFE is primarily driven by the enhancement of its polar component, accompanied by a slight decrease in the dispersive component. Similar to the modification of TPS using the corona discharge method, these changes result from the formation of polar groups in the surface layer (SL) of TPS, which strongly interact with water molecules, thereby improving wettability.

### 3.2. X-Ray Photoelectron Spectroscopy

**Figure 7:**
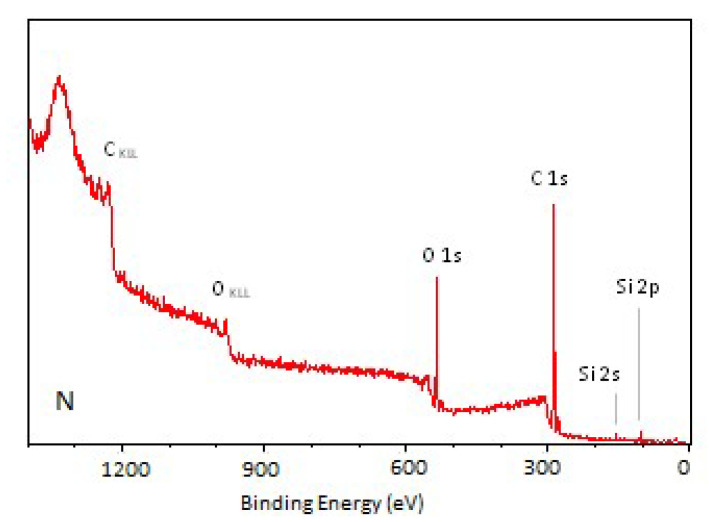
XPS general spectrum of the N sample.

**Figure 8:**
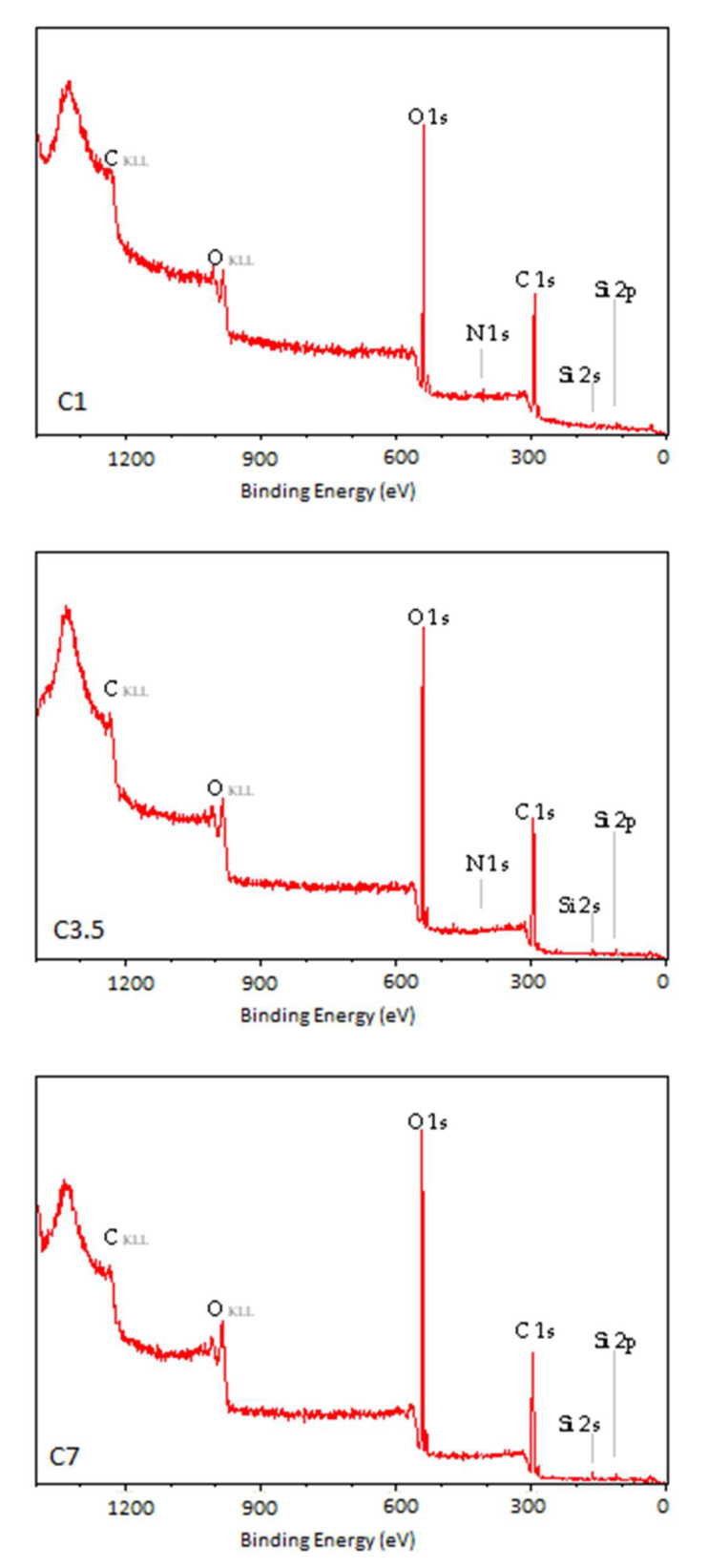

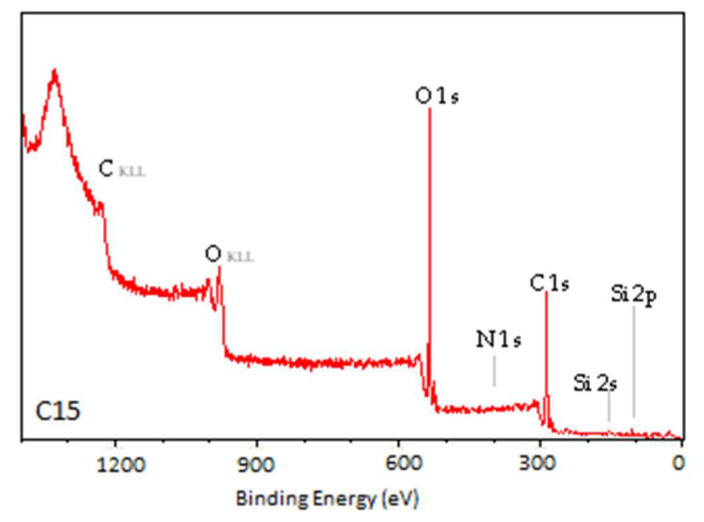
XPS general spectrum of the C1, C3.5, C7, and C15 samples.

**Figure 9:**
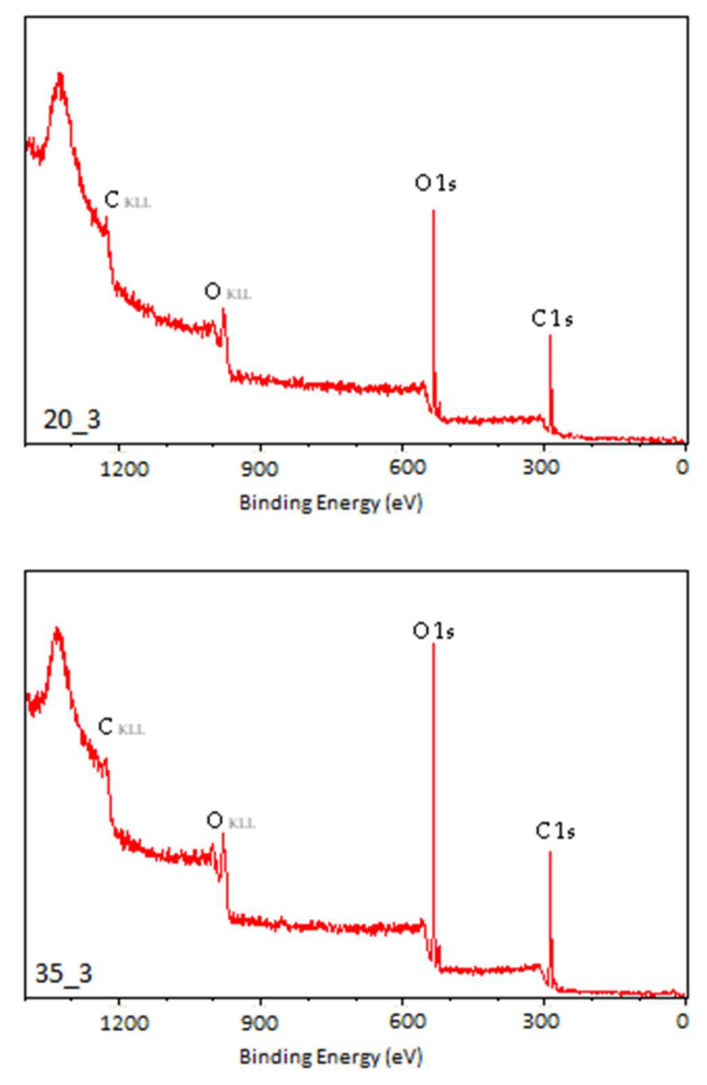

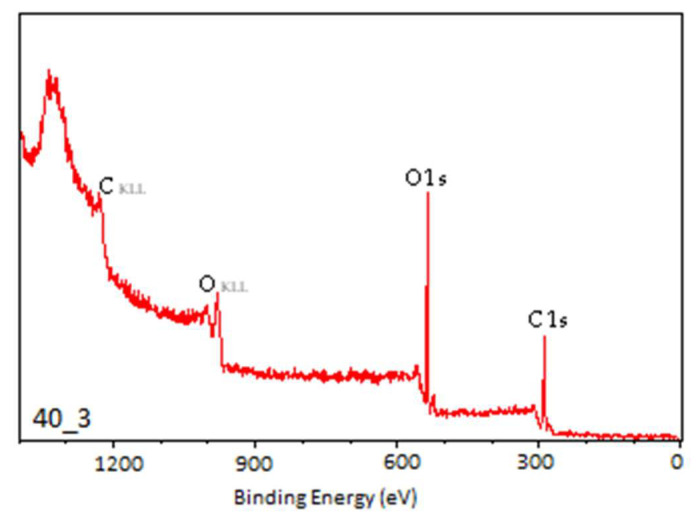
XPS general spectrum of the P20_3, P35_3, and P40_3 samples.

The photoelectron energy distribution spectra of the TPS samples are shown in Figure 7, Figure 8 and Figure 9. Characteristic peaks corresponding to photoelectrons from the internal orbitals of carbon (C_1s_) and oxygen (O_1s_) atoms, as well as trace amounts of silicon (Si_2s_, Si_2p_) and nitrogen (N_1s_), can be observed. These spectra provide the basis for identifying the types of atoms present in the WW of the tested TPS film. The photoelectrons are accompanied by Auger electrons (peaks C_KLL_ and O_KLL_ in the figures), which are emitted from the samples during the tests. Unlike photoelectron energy, the energy of Auger electrons does not depend on the energy of the X-ray radiation incident on the samples. By varying the X-ray energy, the positions of the photoelectron peaks change, while the Auger electron peaks remain at the same position.

The presented spectra indicate an increase in the proportion of oxygen atoms in the WW of the modified TPS samples compared to their proportion in the N sample, which corresponds to a higher degree of oxidation of the WW.

**Figure 10:**
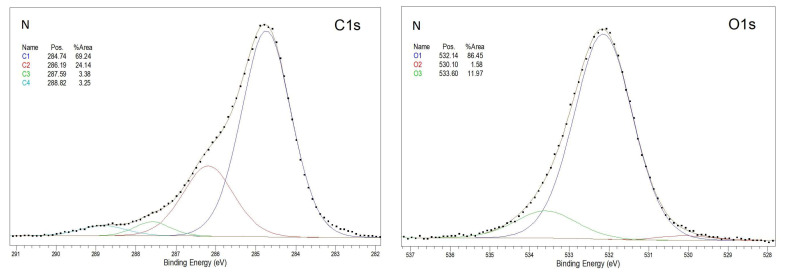

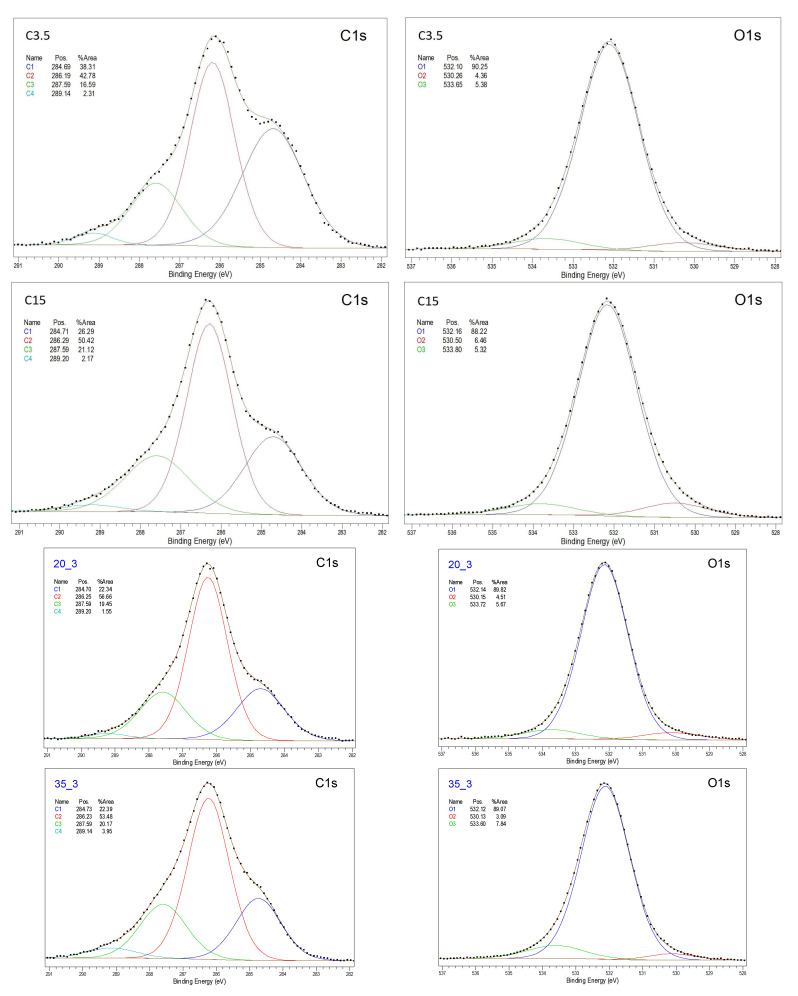

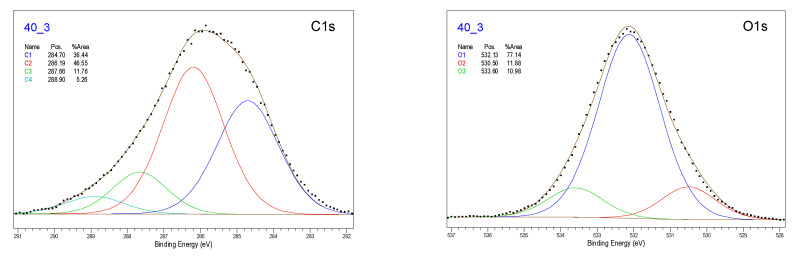
Detailed XPS C1s and O1s spectra of the research samples.

Figure 10 shows the qualitative analysis of functional groups in the TPS surface layer, based on detailed spectra of the C1s and O1s peaks. The peak corresponding to photoelectrons from the C1s level was fitted with four components. Individual peak components corresponding to carbon atoms of different functional groups are marked with colors: blue, red, green, or turquoise. Analogously, the peaks assigned to oxygen atoms of different functional groups are marked with blue, red, or green. The first component, labeled C1 (blue), appears at approximately 284.7 eV and corresponds to graphite-like C–C bonds as well as C–H bonds. The C2 peak (red), observed at around 286.2 eV, is associated with C–O bonds. The C3 peak (green), recorded at approximately 287.6 eV, originates from photoelectrons of the C=O group. The peak labeled C4 (turquoise), detected at around 289.0 eV, most likely corresponds to photoelectrons from the O–C=O group.

The oxygen level was fitted with three components. The most intense peak, labeled O1 (blue), appears at approximately 532.1 eV and most likely originates from C–O or C–OH bonds. The O2 peak (red), observed at around 530.3 eV, is likely associated with C=O groups, while the O3 peak (approximately 533.6 eV, green) may originate from water molecules or chemically adsorbed O_2_ molecules on the sample surface.

The spectral analysis showed that the N sample exhibited the largest content of carbon atoms characterized by a binding energy close to 285 eV. This fraction decreased with increasing energy or modification power. At the same time, the oxygen content in the surface layer of the tested TPS samples increased (Table 4).

The percentage of carbon atoms in the unmodified TPS was 80.49% and decreased to 59.71% and about 60% in the C15 and P_3 samples, respectively, whereas the percentage of oxygen atoms in the unmodified TPS was 16.86% and increased to 37.77% and about 38% in the C15 and P_3 samples, respectively. Additionally, the oxidation degree (O/C) of the samples was calculated, defined as the ratio of the number of oxygen to carbon atoms present in the analyzed WW, expressed as a percentage (Table 5). Small quantities of nitrogen and silicon atoms were also detected, but their concentrations were negligible. As a result, the spectra presented do not show peaks corresponding to these elements. The presence of nitrogen and silicon in the surface layer of the TPS samples may be attributed to lubricants used during the extrusion of the TPS film.

The results of studies on changes occurring in the TPS surface layer under modification (CD and low-pressure plasma under reduced pressure treatment) indicate that, in both cases, the modification led to the formation of C–C, C–O, C=O, and O–C=O groups. The oxygen binding process with TPS induced by low-temperature plasma under reduced pressure proceeds faster than under CD treatment. The oxygen content in the TPS surface layer modified by low-temperature plasma at the lowest applied power (20 W, 3 min) reached 39.43%, whereas, in the case of CD treatment at the lowest energy dose (E_j_ = 1 kJ/m^2^), it reached 26.04%.

The introduction of oxygen atoms into the molecular structure of TPS induces the formation of a dipole moment in macromolecular fragments, thereby increasing the polarity of the surface layer (SL). The physical origin of this dipole moment lies in the significant difference in electronegativity between oxygen atoms and carbon or hydrogen atoms. As a result of this disparity, the electron density involved in bonding oxygen atoms to carbon or hydrogen is higher in the vicinity of oxygen atoms than around carbon or hydrogen atoms. Consequently, the asymmetric distribution of electric charges renders oxygen-containing functional groups with carbon or hydrogen polar in nature. Macromolecules containing polar groups alter the surface properties of TPS, contributing to an increased degree of oxidation.

The degree of oxidation (O/C) of TPS film increases with increasing E_j_ value and plasma power. The increase in the O/C ratio is caused by the formation of oxygen-containing groups, which is initiated by free radicals generated under the influence of electron energy. These electrons strike the modified PLA and subsequently react with oxygen.

### 3.3. The Surface Morphology

**Figure 11:**
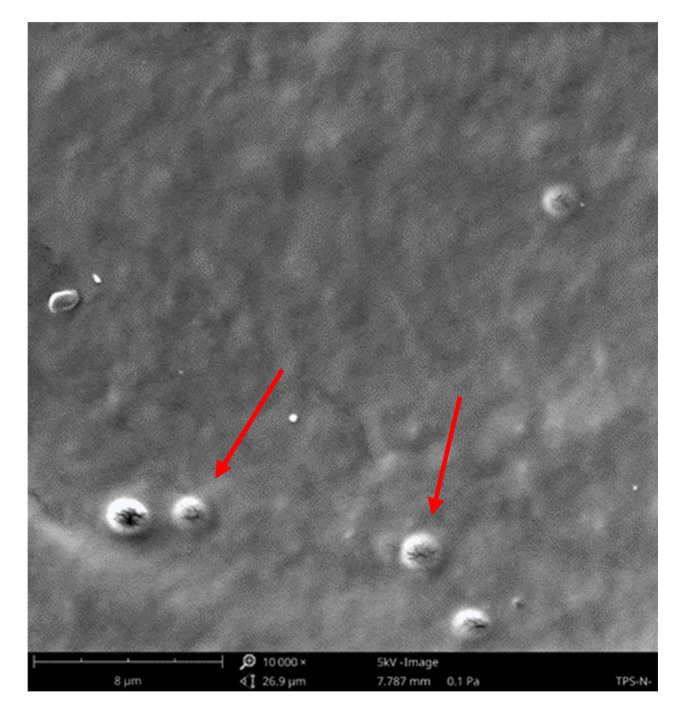
SEM image of the N sample.

Since plasma modification occurs in the WW layer with a thickness of up to 1 μm, depending on the plasma parameters (such as plasma generation power or exposure time), an SEM and a VHX-X1 digital microscope were used to image the TPS surface. Figure 9 and Figure 10 show SEM images of samples modified with plasma under reduced pressure. On the surface of the unmodified film (Figure 11), whole native starch grains can be observed, which were not destructurized during processing (see Figure 11 red arrows).

**Figure 12:**
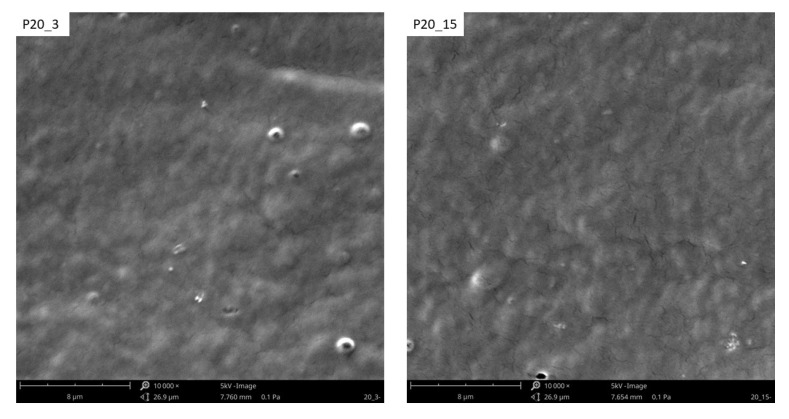

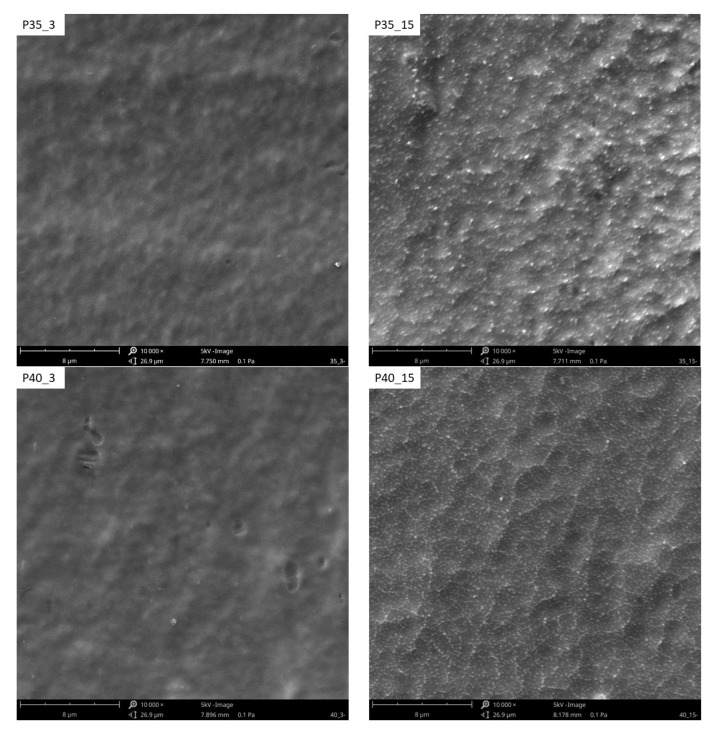
SEM images of the modified TPS film modified with low-temperature plasma.

Plasma modification induces changes in the surface morphology and roughness of the TPS film. Fragment detachment from the surface layer occurs due to ablation, a direct consequence of plasma treatment. As shown in Figure 12, an increase in plasma power and modification time leads to the etching of TPS surface layer fragments (ablation effect). Plasma ablation reactions occur primarily due to the interaction of atomic oxygen, singlet oxygen, or ozone with the modified material—in this case, the TPS film. This process proceeds in two stages. Initially, oxygen-containing functional groups such as –C=O, -C(R)-O-H, and -C(R)-O-O-H form within the surface layer. Subsequently, continued plasma exposure leads to the generation of H_2_O and CO_2_ molecules, which transition into the gaseous state. Ablation may also be initiated by oxygen inherently present in the chemical structure of TPS or by oxygen diffusion between its macromolecules.

**Figure 13:**
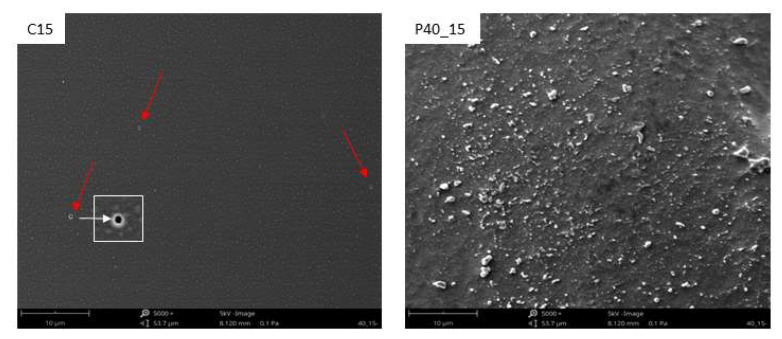
SEM images of the modified TPS film (difference in effects).

In both methods used, more intensive modification led to the formation of degradation products—low-molecular-weight oligomers—on the surface of the TPS film. These oligomers temporarily formed a layer of viscous liquid, resulting in characteristic droplet formation on the TPS surface (Figure 13).

In contrast to low-temperature plasma, corona discharges do not induce significant changes in the geometric structure of the TPS film surface. The primary difference in their effects lies in the formation of streamers—branched plasma channels—during corona discharge modification, leading to uneven surface modification. In Figure 13, for C15, in addition to a thin layer of oligomers, areas of deeper degradation (burnt holes, marked with red arrows in Figure 13) caused by plasma streamers can be observed.

### 3.4. Plasma Sterilization

The reductions (R) in the bacterial strains *Escherichia coli*, *Staphylococcus aureus*, and *Salmonella enteritidis* for the tested samples are shown in Table 6. The results indicate that the corona discharges and low-temperature plasma under reduced pressure are active biocides against bacteria if the modification conditions are appropriate. The effectiveness of modification also depends on the type of bacteria with which we are dealing.

According to the ISO 22196, a biocidal factor is considered effective if R ≥ 2. This study demonstrated that plasma treatment (under appropriate conditions) effectively eliminated bacterial strains of *Escherichia coli*, *Staphylococcus aureus*, and *Salmonella enteritidis*. Selected results meet the requirements of the ISO 22196, confirming that corona discharges and plasma under reduced pressure can be considered effective biocidal factors.

**Figure 14:**
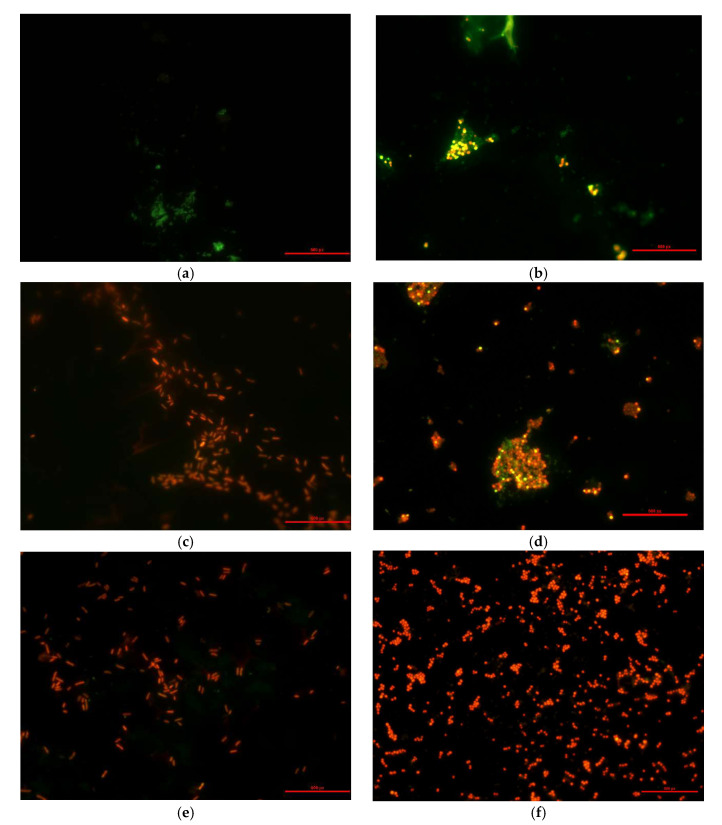
Microscopic images of the TPS film samples with the deposited and stained bacteria of the *Salmonella enteritidis*: (**a**) N, (**c**) C7, (**e**) C15, and *Staphylococcus aureus* strain: (**b**) N, (**d**) C7, (**f**) C15.

Selected microscopic images of stained bacteria from the tested strain, deposited on TPS film specimens, are shown in Figure 14. Microscopic observations revealed that, regardless of the E_j_ value, most *Escherichia coli* and *Salmonella enteritidis* cells were dead on all specimens, except for the non-modified film (N). In samples where R = 1, a fraction of *Staphylococcus aureus* cells remained alive. However, an increase in the E_j_ value or plasma modification power led to a further reduction in the number of viable bacterial cells. For R = 2, all bacterial cells were dead.

The susceptibility of bacteria to plasma as a sterilization method depends on several factors, including the structure of their cell wall, the presence of spores, and defense mechanisms. Gram-negative bacteria, such as *Escherichia coli* and *Pseudomonas aeruginosa*, are more susceptible to plasma treatment. These bacteria have a thin peptidoglycan layer in their cell wall and an outer membrane rich in lipopolysaccharides, which are more vulnerable to damage caused by reactive oxygen species generated in plasma. Plasma disrupts the cell membrane, leading to the leakage of cytoplasmic components and cell death [13].

In contrast, Gram-positive bacteria (e.g., *Staphylococcus aureus*) are less susceptible to plasma treatment due to their thicker peptidoglycan layer, which acts as a protective barrier against oxidative damage. Similarly, spore-forming bacteria (e.g., *Bacillus subtilis*) exhibit increased resistance, as their spores are highly resilient structures capable of withstanding extreme conditions, including UV radiation, desiccation, and reactive oxygen species. Spores possess a thick protective coat and exhibit low metabolic activity, making their elimination by plasma more challenging [14,59].

For each TPS and used plasma method, the inhibition of the ability of microbial spores to reproduce and to cause contamination probably occurs step-by-step and results from the activity of UV photons successively protecting layers of spores and damaging the spores’ DNA material [27].

Apart from this, the morphological evolution induced by plasma treatment induces significant morphological changes on TPS film surfaces, including nano-roughening, microstructuring, and localized etching, which are closely related to modifications in surface properties [38,60,61]. The development of nanostructures on treated surfaces can lead to enhanced antimicrobial activity by two main mechanisms: mechanical disruption of bacterial membranes and inhibition of bacterial adhesion due to reduced effective contact areas [62]. The combination of plasma-induced chemical functionalization and morphological evolution thus provides a synergistic effect that significantly improves the bactericidal performance of treated TPS films.

## 4. Conclusions

The plasma modification of the surface-layer properties of biodegradable materials, particularly packaging films, is an essential technological operation. Its purpose extends beyond merely cleansing and increasing the surface roughness of the modified products; it also involves altering the physical and chemical structure of the surface layer and enhancing surface free energy. Structural modifications primarily include reinforcement through crosslinking processes and the generation of polar functional groups within the surface layer.

Corona discharges and reduced-pressure plasma cause changes in the surface layer of the modified TPS film, including improved wettability and a more than 30% increase in the O/C ratio compared to unmodified TPS film. The polarity of the surface layer increases with the degree of oxidation, which is due to the formation of polar functional groups. This increased oxidation and improved wettability are desirable effects of the modification, as they enhance the adhesion properties of TPS. Plasma treatment also alters the surface morphology and roughness of the TPS film. It induces an ablation process, which causes fragments to detach from the surface layer.

Treatment with low-temperature plasma (under either atmospheric or reduced pressure) demonstrates that these methods can be applied to TPS film sterilization. Plasma may also serve as a supplementary factor in sterilization processes conducted using other physical or chemical methods.

Nevertheless, to validate the potential biomedical applications of modified TPS films, future research should focus on in vivo biocompatibility testing. This could involve subcutaneous implantation in animal models to assess tissue response, as well as evaluating biodegradability under physiological conditions and potential cytotoxicity. These studies will help to determine whether the surface-modified TPS films are suitable for biomedical applications, such as wound dressings or drug delivery systems.

Biodegradable polymers are an innovative material in the field of packaging. Therefore, efforts have been made to develop favorable modification parameters for their surface layer. Biodegradable materials, such as thermoplastic starch (TPS) films, are expected to experience a substantial increase in usage within the packaging industry and disposable products, as starch-based films are renewable, inexpensive, and readily available. For packaging materials, the most crucial aspects are surface hydrophobicity and biocidal properties.

## Figures and Tables

**Table 1:** Modification conditions.

Sample	E_j_ [kJ/m^2^]	v [m/min]
C1	1	96
C2	2	48
C3.5	3.5	27.4
C5	5	19.2
C7	7	13.7
C10	10	9.6
C15	15	6.4

**Table 2:** Symbols of TPS samples and modification conditions.

Sample	P [W]	t [min]
P20_3	20	3
P20_10	20	10
P20_15	20	15
P35_3	35	3
P35_10	35	10
P35_15	35	15
P40_3	40	3
P40_10	40	10
P40_15	40	15

**Table 3:** Conditions for contact angle measurements.

Test Liquid	V [μL]	ΔV [μL/min]	τ [s]
Water	3–7	5	0.5
Diiodomethane	1–3	5	0.5

**Table 4:** Content of carbon and oxygen atoms in the surface layer of the tested TPS samples.

Sample	C1	C2	C3	C4	O1	O2	O3
N	55.73	19.43	2.72	2.61	14.57	0.27	2.02
C1	35.20	24.02	8.72	1.65	22.57	1.37	2.10
C3.5	23.88	26.67	10.34	1.44	31.68	1.53	1.89
C7	19.77	27.53	10.33	2.70	34.20	0.99	2.12
C15	15.70	30.10	12.61	1.30	33.32	2.44	2.01
P20_3	13.53	34.32	11.78	0.94	35.42	1.78	2.23
P35_3	13.98	33.39	12.59	2.47	33.46	1.16	2.95
P40_3	22.80	29.11	7.36	3.29	28.88	4.45	4.11

**Table 5:** The percentage and degree of oxidation (O/C) of tested samples.

Sample	%O	%C	%Si	%N	O/C [%]
N	16.86	80.49	2.65	0.00	20.94
C1	26.04	69.59	3.39	0.98	37.41
C3.5	35.10	62.33	2.16	0.41	56.31
C7	37.31	60.33	2.36	0.00	61.84
C15	37.77	59,71	1.96	0.56	63.25
P20_3	39.43	60.57	0.00	0.00	60.09
P35_3	37.57	62.43	0.00	0.00	60.17
P40_3	37.44	62.56	0.00	0.00	59.84

**Table 6:** The reductions (R) in bacterial strains.

Sample	*Escherichia coli*	*Staphylococcus aureus*	*Salmonella enteritidis*
N	0	0	0
C1	2	1	2
C3.5	2	1	2
C7	3	1	3
C15	3	2	3
P20_3	1	1	2
P35_3	1	1	2
P40_3	2	3	3

## Data Availability

Data are contained within the article.
